# Comparison of single and combination diuretics on glucose tolerance (PATHWAY-3): protocol for a randomised double-blind trial in patients with essential hypertension

**DOI:** 10.1136/bmjopen-2015-008086

**Published:** 2015-08-07

**Authors:** Morris J Brown, Bryan Williams, Thomas M MacDonald, Mark Caulfield, J Kennedy Cruickshank, Gordon McInnes, Peter Sever, David J Webb, Jackie Salsbury, Steve Morant, Ian Ford

**Affiliations:** 1 Clinical Pharmacology Unit, Addenbrooke's Hospital, University of Cambridge, Cambridge, UK; 2 Trial Executive for the British Hypertension Society's PATHWAY Programme of Trials; 3 Steering Committee for the British Hypertension Society's PATHWAY Programme of Trials; 4 Institute of Cardiovascular Sciences, University College London, London, UK; 5 Medicines Monitoring Unit, Medical Research Institute, University of Dundee, Dundee, Tayside, UK; 6 William Harvey Institute, QMUL, London, UK; 7 Cardiovascular Medicine & Diabetes, King's College London, London, UK; 8 Institute of Cardiovascular Medical Sciences, Western Infirmary, London, UK; 9 Centre of Circulatory Health, Imperial College, London, UK; 10 Clinical Pharmacology Unit, University of Edinburgh, Edinburgh, UK; 11 Robertson Centre, University of Glasgow, Glasgow, UK

**Keywords:** CLINICAL PHARMACOLOGY

## Abstract

**Introduction:**

Thiazide diuretics are associated with increased risk of diabetes mellitus. This risk may arise from K^+^-depletion. We hypothesised that a K^+^-sparing diuretic will improve glucose tolerance, and that combination of low-dose thiazide with K^+^-sparing diuretic will improve both blood pressure reduction and glucose tolerance, compared to a high-dose thiazide.

**Methods and analysis:**

This is a parallel-group, randomised, double-blind, multicentre trial, comparing hydrochlorothiazide 25–50 mg, amiloride 10–20 mg and combination of both diuretics at half these doses. A single-blind placebo run-in of 1 month is followed by 24 weeks of blinded active treatment. There is forced dose-doubling after 3 months. The *Primary end point* is the blood glucose 2 h after oral ingestion of a 75 g glucose drink (OGTT), following overnight fasting. The primary outcome is the difference between 2 h glucose at weeks 0, 12 and 24. *Secondary outcomes* include the changes in home systolic blood pressure (BP) and glycated haemoglobin and prediction of response by baseline plasma renin. Eligibility criteria are: age 18–79, systolic BP on permitted background treatment ≥140 mm Hg and home BP ≥130 mm Hg and one component of the metabolic syndrome additional to hypertension. Principal exclusions are diabetes, estimated-glomerular filtration rate <45 mL/min, abnormal plasma K^+^, clinic SBP >200 mm Hg or DBP >120 mm Hg (box 2). The sample size calculation indicates that 486 patients will give 80% power at α=0.01 to detect a difference in means of 1 mmol/L (SD=2.2) between 2 h glucose on hydrochlorothiazide and comparators.

**Ethics and dissemination:**

PATHWAY-3 was approved by Cambridge South Ethics Committee, number 09/H035/19. The trial results will be published in a peer-reviewed scientific journal.

**Trial registration numbers:**

Eudract number 2009-010068-41 and clinical trials registration number: NCT02351973.

**Strengths and limitations of this study**

- This is a parallel-group, randomised, double-blind, multicentre trial.
- Six months may not be sufficient to permit detection of new onset diabetes.
- Two of the randomised treatments are available in the UK only in combination formulations containing different doses from those under study.

## Introduction

Thiazide and thiazide-like diuretics are widely used. However, such diuretics are associated with increased risk of developing diabetes mellitus.^1^ This risk may arise from K^+^-depletion and be avoided by use of K^+^-sparing diuretics. We therefore hypothesised that a K^+^-sparing diuretic has a beneficial influence on glucose tolerance compared to a thiazide, and that the use of low-dose thiazide combined with a K^+^-sparing diuretic may achieve similar blood pressure reduction, but improved glucose tolerance, compared to a high-dose thiazide.

**Figure 1:**
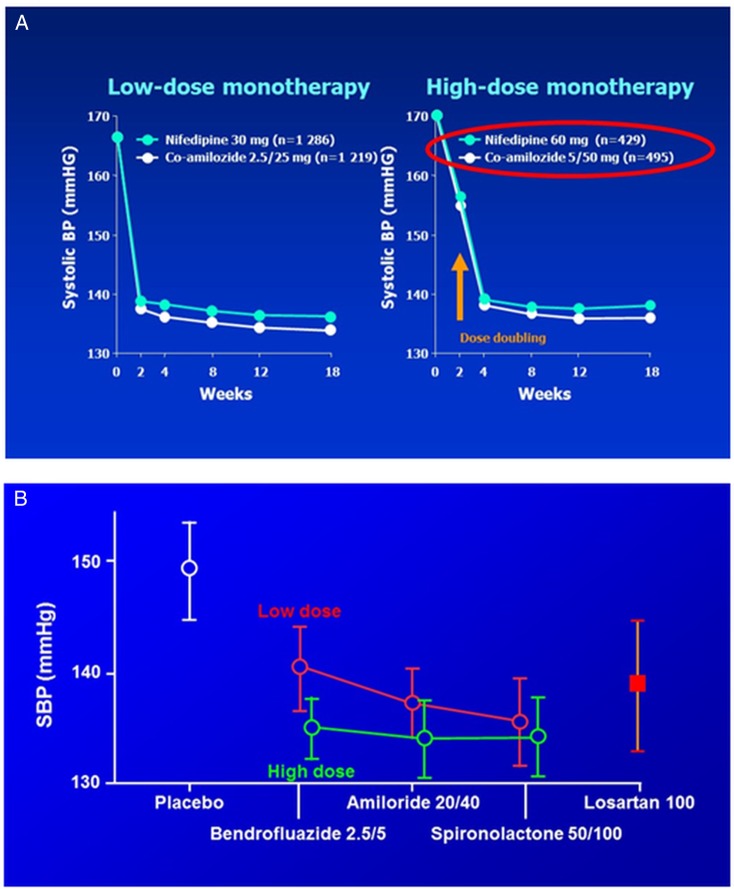
Evidence for dose–response to thiazide diuretics (A). Comparison of blood pressure response to dose-doubling of a calcium-channel blocker, nifedipine and diuretic combination, hydrochlorothiazide and amiloride, in the INSIGHT study. The figure shows the response in patients achieving target blood pressure, 140/90 mm Hg, on low-dose (left panel) or high-dose (right panel) monotherapy. (Unpublished data from reference 6) (B). Comparison of blood pressure response to dose-doubling of three types of diuretic-bendroflumethiazide, amiloride, spironolactone. (Data redrawn from ref. 8).

Diuretics are no longer used at doses achieving maximum reduction in BP. This is because of the evidence that higher doses are associated with increased risk of diabetes mellitus (DM), and an extrapolation from small studies in the 1980's and 90's that maximal blood pressure reductions were achieved by low-dose thiazides.^2–5^ However, unpublished dose-titration data from INSIGHT (figure 1A), where the average age was 60, shows as steep a dose–response for hydrochlorothiazide as for nifedipine in patients (right panel) whose dose was doubled after 2 weeks.^6^ Amiloride has never been fully investigated at doses equi-effective with thiazides, and is used mainly in an ancillary K^+^-sparing role.^7^ A diuretic crossover study (‘SALT’) confirmed that in low-renin patients bendroflumethiazide 2.5 mg is not maximal, and showed either spironolactone or amiloride to be effective alternatives to the higher dose (figure 1B).^8^ Several indices in SALT indicated that even 5 mg of bendroflumethiazide was a less effective natriuretic than the K^+^-sparing diuretics, perhaps because it lowers blood pressure (BP) partly through vasodilation.^9^ The difference in mechanisms raised the possibility, to be explored by PATHWAY-3, that the diuretics will be found to have an additive effect on BP.

**Figure 2:**
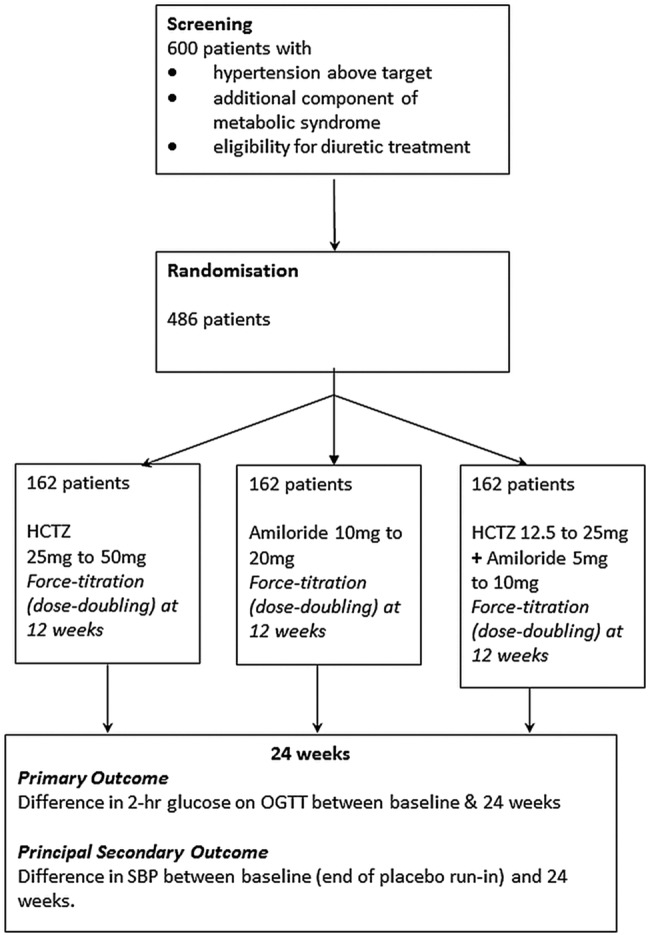
Trial schematic.

*Diuretics and new-onset diabetes*: A major attraction of K^+^-sparing diuretics is the possibility that they will offset the diabetogenic potential of thiazides. Since they have not been compared in hypertension outcome trials, and diabetes (DM) has not been an end point in heart failure studies of spironolactone or eplerenone, we do not know for certain whether they are clean in this respect. Short-term studies suggest they are.^10^ Interestingly in INSIGHT there was no excess of DM in patients receiving hydrochlorothiazide (HCTZ) 25 mg, which was combined with amiloride 2.5 mg, but increased by 30% in patients on HCTZ/amiloride 50/5 mg.^6^ In PATHWAY-3, we use the oral glucose tolerance test (OGTT) to provide an end point for each subject. This strategy was previously used to demonstrate a difference after just 12 weeks of dosing with a thiazide diuretic.^11^ In the STAR study, which compared 200 markedly obese patients randomly assigned to either ACE inhibitor +Ca^++^ blocker, or ARB+thiazide, the subjects had impaired glucose tolerance at entry, allowing detection of changes on low-dose thiazide. Subsequently, two small crossover studies in about 40 patients showed a rise in 2 h glucose within 4 weeks of treatment with bendroflumethiazide 5 mg or HCTZ 50 mg, with a highly significant difference from the 2 h glucose during 4 weeks of treatment with amiloride 20 mg (figures 1 and 2).^12^

PATHWAY-3 will test whether the apparent superiority of amiloride, in protecting glucose tolerance, is maintained over 6 months of treatment, and translates into measurable differences in HbA1c. The study will be large enough for secondary estimates of mechanism, for example, the 0 and 30 min plasma insulin, to determine whether the main drug effects are on insulin secretion or sensitivity, and are influenced by the opposite effects of the two diuretics on plasma K^+^. However, owing to the lack of long-term study of amiloride other than in combination with HCTZ, the study is also evaluating a group in which the two diuretics are used in combination. If we demonstrate that HCTZ 25 mg+amiloride 10 mg achieves the same (or greater) blood pressure reduction as HCTZ 50 mg, without an adverse effect on OGTT, this could become the recommended diuretic treatment for hypertension in the future.

In order to maximise recruitment, while also maximising sensitivity to detect changes in OGTT, the trial is open to most of those patients with hypertension in whom diuretic is a reasonable next option, providing they have one feature of the metabolic syndrome—additional to hypertension. This broad eligibility allows us also to assess safety of amiloride in combination with all commonly used antihypertensive drugs.

The initial protocol was approved by the Medicines and Healthcare Products Regulatory Agency (MHRA) on 8 May 2009, and is visible at https://www.clinicaltrialsregister.eu/ctr-search/trial/2009-010068-41/GB#A. This was not registered until 2015, because of the prior registrations with MHRA and UKCRN and local advice that these sufficed. The current protocol is V.8, as approved on 13 February 2013. Any further amendments will be approved by Research Ethics and MHRA and registered also with clinicaltrials.gov.

### Primary objectives

The primary objective of the study is to determine whether a K^+^-sparing diuretic can be safely substituted for, or combined with, a thiazide diuretic in order to maximise the long-term benefits of diuretic treatment.

### Secondary objectives

The secondary objectives of the study are:

- To demonstrate whether half-dose combination of two classes of diuretic improve efficacy and tolerability of diuretics, compared to taking one class alone.
- To evaluate the mechanism of changes in glucose tolerance, particularly whether these are related to changes in K^+^.
- To determine whether a baseline measurement of plasma renin, measured on various background treatment permutations predicts whether patients’ blood pressure is likely to be improved by addition of either low-dose or high-dose diuretic
- To determine the best predictors of patients whose glucose tolerance will be impaired by addition of thiazide diuretic.

A further secondary objective is to establish a repository of pharmacogenetic samples and investigate relationships between genetic factors and pharmacodynamic responses.

## Methods and analysis

### Trial design

#### Overall trial design

This is a parallel-group, randomised, double-blind, multicentre trial, comparing three treatment strategies in patients with hypertension, an indication for diuretic treatment, and at least one other component (ie, additional to hypertension) of the metabolic syndrome. Following a month's placebo run-in, patients receive their randomised treatment (diuretic) in addition to existing background therapy for 6 months, with an OGTT at the beginning, middle and end of this period. The dose of each diuretic is doubled after the second (3-month) OGTT. The trial design is outlined in the flow chart (figure 2).

### Study population

Inclusion criteria are shown in box 1. These are intended to enable recruitment of most patients in whom addition of diuretic might be part of usual practice, enriched for patients most likely to be at risk of develop type 2 DM during long-term treatment with thiazide diuretic.

Box 1 **Inclusion criteria**

1. Age 18-80
2. Diagnosis of hypertension according to BHS criteria
3. Systolic blood pressure (SBP) on permitted background treatment ≥140 mm Hg and home BP ≥130 mm Hg.
4. Indication for diuretic treatment as a treatment option for the patient's uncontrolled hypertension:
  - Untreated + (age >55 AND/OR Black AND/OR renin <12 mU/L)
    OR
  - Receiving one or any permutation of the following:
    ACEi, ARB, β-blocker, CCB, direct renin inhibitor
5. At least one other component (ie, additional to hypertension) of the metabolic syndrome (reduced high-density lipoprotein (HDL), raised triglycerides, glucose, waist circumference)*

*Definition of Metabolic Syndrome according to the International Diabetes Federation, 2006:

Central obesity (waist circumference >94 cm male (>90 if Asian), >80 female plus two of:

- SBP ≥130 or diastolic blood pressure ≥85 mm Hg
- Fasting glucose >5.6 mmol/L
- Fasting triglycerides >1.7 mmol/L (or on treatment)
- HDL <1.03 mmol/L males, <1.29 mmol/L females (or on treatment)

The PATHWAY programme anticipated changes to the definition of hypertension introduced by the National Institute for Health and Care Excellence (NICE) guidance of 2011. The trials use home BP measurements as an outcome measure, and patients are required to exceed threshold levels of both clinic BP (at screening and/or randomisation) and home BP (at randomisation). Initially we set the clinic threshold at 145 mm Hg, until we had enough experience within the trial of adding high-dose hydrochlorothiazide or amiloride to multiple background drugs. The threshold was then reduced, to 140 mm Hg (see box 1).

### Recruitment and randomisation of participants

Potentially suitable patients are identified from hospital and general practice populations. Written informed consent is obtained from participants by a medical investigator. The research nurse records baseline variables, takes blood and urine for baseline biochemistry and haematology and records the medical history. Blood samples are analysed at the local health service laboratory according to usual practice. Serum for future analyses and blood for future genetic analyses are stored by centres. Subjects who have given informed consent, and meet the inclusion and exclusion criteria at the end of a month's placebo run-in, are randomised to receive hydrochlorothiazide 25 mg daily, amiloride 10 mg daily, or a combination of hydrochlorothiazide 12.5 mg and amiloride 5 mg daily, each in addition to any other antihypertensive drug being taken at the time of randomisation. Randomisation is performed by contacting a central computerised randomisation facility based at the Robertson Centre for Biostatistics, University of Glasgow by telephone or via a web-based service.

### Trial treatments

Initial treatment is the three groups described above. After 3 months, each of the groups are force-titrated to twice the starting dose, namely hydrochlorothiazide 50 mg daily, amiloride 20 mg daily, or a combination of hydrochlorothiazide 25 mg and amiloride 10 mg daily. These three groups are shown in the flow diagram (figure 2). Trial medication is provided in identical-looking containers for each of the three assignments by the Royal Free Hospital Pharmacy, and labelled without use of the drug name, according to a randomisation schedule provided by the Robertson Centre, University of Glasgow. None of the investigators, patients or laboratory staff undertaking the primary outcome measures are informed of the assignment. A 24 h telephone unblinding service is provided by the Data Management Centre for instances where principal investigators believe that treatment of an adverse event may be compromised by their not knowing treatment assignment. Compliance has been assessed by returned tablet counts.

### Tolerability

Adverse events are recorded in the electronic case record form at each visit. A 2-week drug holiday is permitted at any point where the investigator considers this may allow subjects to remain in the trial without early withdrawal.

Box 2 **Exclusion criteria**

1. Diabetes (types 1 or 2)
2. Secondary hypertension
3. Estimated-glomerular filtration rate < 45 mLs/min
4. Plasma K^+^ outside normal range on two successive measurements during screening
5. Clinic systolic blood pressure >200 mm Hg or diastolic blood pressure >120 mm Hg, with principal investigator (PI) discretion to override if home BP measurements are lower
6. Requirement for diuretic therapy (other than for hypertension)
7. Absolute contraindications to any of the study drugs (listed on their data sheet)
8. Current therapy for cancer
9. Anticipation of change in medical status during course of trial (eg, planned surgical intervention requiring >2 weeks convalescence, actual or planned pregnancy)
10. Inability to give informed consent
11. Not on stable doses of all hypertensive medications to be continued throughout the study for a minimum of 4 weeks prior to randomisation, or not normally less than 2 weeks if early randomisation is required at the discretion of the PI.
12. Participation in a clinical study involving an investigational drug or device within 4 weeks of screening.
13. Any concomitant condition that, in the opinion of the investigator, may adversely affect the safety and/or efficacy of the study drug or severely limit the subject's lifespan or ability to complete the study (eg, alcohol or drug abuse, disabling or terminal illness, mental disorders).
14. Treatment with any of the following prohibited medications:
  - Oral corticosteroids within 3 months of screening.
  - Chronic use (defined as ≥3 days of treatment per week) of non-steroidal anti-inflammatory drugs (NSAIDs) other than acetylsalicylic acid.
  - The use of short-acting oral nitrates within 4 h of screening or any subsequent study visit; long-acting oral nitrates (eg, Isordil) is permitted, but the dose must be stable for at least 2 weeks prior to screening and randomisation.
15. A pill count will be made at the end of the 4-week run-in period and those with adherence <70% will be excluded from randomisation

### Trial procedures

These are shown for each visit in the schedule of assessments (table 1). There are three principal visits, at 0, 12 and 24 weeks, at which subjects have an OGTT. Blood glucose is measured at 0, 30, 60, 120 min and insulin at 0 and 30 min. At these visits, blood is also collected for electrolytes and estimated-glomerular filtration rate (eGFR), plasma renin, glycated haemoglobin (HbA1c) and plasma lipids. Electrolytes and eGFR are also checked at 2 and 14 weeks, namely 2 weeks after initiation and dose-doubling of trial diuretic medication. Seated home blood pressure readings are recorded (morning and evening, in triplicate) over 4 days prior to each of the three principal visits, using the Microlife WatchBP monitor. Clinic blood pressure is measured in triplicate at each visit, by the same monitor. For analyses of home blood pressure, we will use the average of the last 18 recordings prior to the visit—that is, from days −1, −2 and −3 if all recordings have been undertaken. For clinic blood pressure, we will analyse the average of readings 2 and 3.

**Table 1:** Schedule of measurements

Assessment	Screening	Placebo run-in D-3, D-2, D-1	Week 0	Week 2	Week 11 D-3, D-2, D-1	Week 12	Week 14	Week 23 D-3, D-2, D-1	Week 24
Informed consent	x								
Demography	x								
Medical history	x		x						
Medical examination	x								
Concomitant medications	x		x	x		x	x		x
Inclusion/exclusion checks	x		x						
Height and weight*	x		x			x			x
Clinic BP†	x		x			x			x
Home BP‡		x			x			x	
ECG	x								
Waist and hip circumference	x								x
Urinalysis	x		x						x
*Blood Tests:*									
Electrolytes (including bicarbonate)	x		x	x		x	x		x
Glucose (non-fasting)	x								
Full-blood count	x					x			x
Lipid profile	x		x			x			x
Uric acid	x					x			x
Ca++	x					x			x
Renin			x			x			x
Pharmacogenetics§			x						
HbA1c			x			x			x
Glucose(fast)¶			x			x			x
Insulin¶			x			x			x
OGTT**			x			x			x
Pregnancy serum††	x								
Adverse events						x			x
Randomisation			x						
Study medication dispensed	x		x			x			
Compliance check			x			x			x
Dose force titrated						x			

*Height recorded at first visit only.

†Clinic BP will be measured following 10 min rest and recorded in triplicate.

‡Home BP will be measured using the BP device given by clinic at approximately 08.00 and 20:00 on the 4 days before the clinic visit. Patients will be asked to take triplicate reading after 10 min rest and to record the second and third on the proforma provided.

§Pharmacogenetics sample to be taken where specific informed consent has been given. Sampling will typically be at baseline (day 0), but may be at any time later in the study.

¶ That is, baseline sample for OGTT.

**Glucose at 0, 30, 60, 120 min; insulin at 0, 30 min.

††Serum HcG may be replaced by early morning urine specimen for human choriogonadotropin testing.

BP, blood pressure; HbA1c, glycated haemoglobin; OGTT, oral glucose tolerance test.

### Trial end points

#### Primary end point

The primary study end point is the difference in blood glucose, measured 2 h after oral ingestion of a 75 g glucose drink, between the final day of the placebo run-in and at the end of 3 months and 6 months of blinded treatment.

### Secondary end points

These are:

- Difference in area under the curve of the OGTT between the final day of the placebo run-in, and at the end of 3 months and 6 months of blinded treatment
- Difference in plasma insulin at 30 min, between the final day of the placebo run-in, and at the end of 3 months and 6 months of blinded treatment
- Difference in fasting serum lipids, between the final day of the placebo run-in, and at the end of 3 months and 6 months of blinded treatment
- The change in home systolic BP from end of placebo run-in to the end of 3 months and 6 months of blinded treatment.
- The change in clinic systolic BP from end of placebo run-in to the end of 3 months and 6 months of blinded treatment.
- The natriuretic response, as assessed from the compensatory increase in plasma renin from end of placebo run-in to the end of 3 months and 6 months of blinded treatment.
- Prediction, by baseline plasma renin, of clinic and home SBP response to each treatment.

### Data handling and record keeping

Study data is recorded by remote data entry into a web-based electronic case report form (eCRF) developed for the study by the Robertson Centre, Glasgow. eCRF data is anonymous and will identify study subjects by their assigned study numbers only. All missing data, possible duplication and data outside pre-set limits for each parameter, is queried by the Management Centre, and will be internally validated before database lock.

### Data analysis

#### Sample size determination

Based on at least 80% power to detect a mean difference in glucose between any two of the treatment arms of 1 mmol/L (SD=2.2 mmol/L) using two-sample t tests with a 1% significance level, 414 patients are required. This is the observed difference in 2 h glucose in the largest previous trial of glucose intolerance caused by HCTZ.^11^ Adjusting for an anticipated dropout proportion of 15%, 486 patients are required overall—162 in each treatment arm.

Analysis will be performed on the full analysis population—defined as all patients with at least one postbaseline visit—on an Intention-To-Treat basis. For sensitivity, all analyses will also be performed on the per-protocol population—defined as all patients with at least one post baseline visit and excluding those with any form of major violation of the study.

Recruitment started in November 2009, and is expected to finish during 2015.

### Statistical plan

In order to meet the primary objective of determining whether amiloride should be substituted for, or added to, hydrochlorothiazide, the study has a hierarchical coprimary end point. The first-tested comparison will be amiloride versus HCTZ. The second tested will be combination versus HCTZ. A mixed effects model will be used to compare the 2 h glucose on OGTT between the three treatment groups (baseline, 12 and 24 weeks). This model will adjust for baseline covariates.

For secondary analyses, the primary analysis will be repeated but with the area under the curve (AUC) of the OGTT as the dependent variable. Mixed effects models will be used to estimate treatment effects for home and clinic SBP, and for HbA1c. Analysis of covariance will be used to compare: insulin (fasting, and rise at 30 min during OGTT), HbA1c, lipid profile, renin mass and weight at the end of study between the three treatment groups adjusting for baseline measures. Logistic models will be used to compare the proportion of subjects to achieve target SBP (defined as ≤140 mm Hg) at 24 weeks between the treatment groups. Logistic models will be used to compare the proportion of patients who develop diabetes (defined by fasting glucose ≥7 mmol/L or 2 h glucose ≥11.1 mmol/L or HbA1c ≥6.5%) by the end of the study between the three treatment groups. The covariates in analyses of BP will include baseline plasma renin as a potential predictor of response.

Patients who withdraw from the study before final visit will be included in the primary analysis if they have at least one postrandomisation glucose tolerance test, and missing data imputed by application of last observation carried forwards. Patients with data missing from any timepoint required for analysis, and patients in whom major violation of the protocol is documented by investigators, or detected by the data management centre, will be excluded from per-protocol analysis.

There will be no interim analysis, no stopping rules and no data monitoring committee. This is because all treatments are being used for licensed indications, and have been so used for several decades. We do not therefore anticipate any unexpected hazard that has eluded detection during many hundreds of thousands of person-years exposure; and the study is not powered to detect any significant differences in serious morbidity or mortality between treatment groups.

### Ethics and dissemination

PATHWAY-3 is approved by Cambridge South Ethics Committee and the MHRA. The results will be published in a peer-reviewed journal, and presented to national and international meetings. All authors of this article will have full access to the complete data set, subject only to agreement by coauthors to uses of the data. Authorship of future articles reporting outcomes will represent multidisciplinary input at each site, with the articles being written by a subset of the current authorship. There are no current plans to make anonymised participant-level data publicly available. However lay-friendly summaries of our findings will be sent to all our patients, and we expect to work with the British Heart Foundation to maximise patient and public access to the findings.

### Ancillary and post-trial care

During the trial all patients are covered by the National Health Service (NHS) indemnity. We expect most patients to continue diuretic treatment in addition to other pretrial background therapy that has been continued during the trial.

### Study sponsorship: monitoring, audit, quality control and quality assurance

The trial is sponsored by the University of Cambridge and Cambridge University Hospitals NHS Foundation Trust, contact stephen.kelleher@addenbrookes.nhs.uk. Trial investigators will permit authorised third parties access to the trial site and medical records relating to trial subjects. This will include, but not necessarily be restricted to, access for trial-related monitoring, audits, Ethics Committee review and regulatory inspections. We do not expect funders or sponsors to be involved in data analysis or reporting.

### Associated projects

This study (PATHWAY-3) is one of three complementary studies in a British Heart Foundation-funded programme which will investigate optimal treatment for patients with hypertension. PATHWAY-1 will investigate whether initial treatment with a combination of drugs is more effective in achieving a sustained target pressure than starting with monotherapy and adding a second drug. PATHWAY-2 will recruit patients with more severe hypertension than either PATHWAY-1 or PATHWAY-3, and compare the blood pressure response to each of the three classes recommended by current guidelines.
